# Effect of Withanolide A on 7-Ketocholesterol Induced Cytotoxicity in hCMEC/D3 Brain Endothelial Cells

**DOI:** 10.3390/cells11030457

**Published:** 2022-01-28

**Authors:** Sandra Soh, Wei-Yi Ong

**Affiliations:** 1 Department of Anatomy, Yong Loo Lin School of Medicine, National University of Singapore, Singapore 119260, Singapore; e0260122@u.nus.edu; 2 Neurobiology Research Programme, Life Sciences Institute, National University of Singapore, Singapore 119260, Singapore

**Keywords:** withanolide A, 7-ketocholesterol, brain endothelial cells, blood-brain-barrier, hypercholesterolemia, diabetes mellitus, vascular dementia, cerebral ischemia

## Abstract

Withanolide A is a naturally occurring phytochemical that is found in Ashwagandha (*Withania somnifera*, fam. Solanaceae) or Indian Ginseng. In the current study, we elucidated the effect of withanolide A on 7-ketocholesterol (7KC) induced injury in hCMEC/D3 human brain endothelial cells. 7KC is a cholesterol oxidation product or oxysterol that is present in atherosclerotic plaques and is elevated in the plasma of patients with hypercholesterolemia and/or diabetes mellitus. Results showed that withanolide A significantly reduced the effects of 7KC, which include loss of endothelial cell viability, increase in expression of pro-inflammatory genes-IL-1β, IL-6, IL-8, TNF-α, cyclooxygenase-2 (COX-2), increased COX-2 enzyme activity, increased ROS formation, increased expression of inducible nitric oxide synthase and genes associated with blood clotting, including Factor 2/thrombin, Factor 8, von Willebrand factor, and thromboxane A synthase, and increased human thrombin enzyme activity. Some of the above effects of withanolide A on 7KC were reduced in the presence of the glucocorticoid receptor antagonist, mifepristone (RU486). These findings suggest that the glucocorticoid receptor could play a role in the cytoprotective, antioxidant, and anti-clotting effects of withanolide A against 7KC. Further studies are necessary to elucidate the detailed mechanisms of action of withanolide A against oxysterol-induced injury.

## 1. Introduction

The Blood Brain Barrier (BBB) maintains ionic compositions needed for neuronal function and prevents central nervous system (CNS) neurons from being exposed to toxins in circulation. Age-related changes could occur in the BBB, however, and accelerated degradation has been reported, in patients with mild cognitive impairment compared to age- matched neurologically intact controls [1]. One of the factors that could lead to injury of brain endothelial cells and damage to the BBB is cholesterol oxidation products or oxysterols. These products are found in fatty streaks and advanced lesions in atherosclerosis [2]. 7-Ketocholesterol (7KC) is the most common product of a reaction between cholesterol and oxygen radicals and is the most concentrated oxysterol found in the blood and arterial plaques of coronary artery disease patients, as well as various other disease tissues and cell types. Unlike cholesterol, 7KC consistently shows cytotoxicity to cells [3]. Total cholesterol oxidation products including 7KC, as well as 7α- and 7β- hydroxycholesterol, are significantly greater in patients with hypertriglyceridemia, hypertension, diabetes and overweight/obesity status compared to those without cardiovascular risk factors [4,5]. 7KC and 7β-hydroxycholesterol cause a specific form of cytotoxic activity defined as oxiapoptophagy, including oxidative stress and induction of death by apoptosis associated with autophagic criteria. Oxiaptophagy is associated with organelle dysfunction—in particular, mitochondrial and peroxisomal alterations involved in the induction of cell death and disruption of redox balance [6]. Oxysterols including 7KC and 7β-hydroxycholesterol show potent cytotoxic effects on bovine aortic endothelial cells. Internucleosomal DNA fragmentations are detected in these cells after 7KC treatment, indicating that the oxysterol induces cell death via apoptosis [7]. 7KC induces a transient increase in intracellular Ca^2+^ levels, production of reactive oxygen species (ROS), and apoptosis in mouse aorta endothelial cells, and antioxidants and calcium antagonists are found to attenuate 7KC-induced cell death [8]. 7KC causes apoptosis in endothelial cells via activation of caspases-8, -12, and -3/7 [9]. Moreover, 7KC induces ROS production, ATM/Chk2, ATR-Chk1 and p53 signaling pathways, G0/G1 cell cycle arrest and apoptosis, and increases the expression and secretion of the inflammatory cytokine IL-8 in endothelial cells [10]. 7KC induced damage results in increased expression of the pro-atherogenic protein profilin-1 in aortic endothelial cells [11] and increased the expression and release of a clotting protein, von Willebrand factor in a human endothelial cell line (HUVEC-CS) [12].

Currently, several natural compounds or mixtures of compounds used in traditional medicine are able to inhibit the deleterious effects of 7KC. The different molecules identified could be used as functional foods to prevent or treat diseases associated with 7KC [13]. Natural molecules and oils, often associated with the Mediterranean diet, as well as synthetic molecules, have proved effective in vitro [14]. Ashwagandha (*Withania somnifera*, fam. Solanaceae) or Indian Ginseng is a common Indian herb used in Ayurvedic medicine as an adaptogen or anti-stress agent to improve health [15]. Recent studies have indicated that Ashwagandha root improves the body’s defense against chronic diseases through its antioxidant and anti-inflammatory effects that protect against cellular damage caused by free radicals and inflammatory mediators [16]. Ashwagandha extracts not only effectively inhibits lipopolysaccharide-induced ROS production in BV-2 microglial cells, but also stimulates the nuclear factor (erythroid-derived 2)-like 2 (Nrf2) pathways, leading to increased antioxidant responses [17]. Chemically, powder from the roots of Ashwagandha contains a large variety of compounds including 12 alkaloids, 40 withanolides and several sitoindosides and flavonoids isolated from different parts of the plant [18,19,20], reviewed in [15]. Withanolide A and Withaferin A are two constituents that show similar pharmacokinetic profiles in this plant [21]. Withanolide A has been found to suppress NF-κB activation induced by a variety of inflammatory and carcinogenic agents, including tumor necrosis factor-α (TNF-α), interleukin- 1β (IL-1β), and doxorubicin [22]. Another study demonstrates an anti-oxidative effect of withanolide A and suggests that it increases glutathione biosynthesis in neuronal cells by upregulating glutamate-cysteine ligase catalytic subunit level through the Nrf2 pathway, in a corticosterone dependent manner [23].

To date, however, little is known about the possible effects of withanolide A on changes induced by oxysterols. This study was therefore carried out, in view of the potential health benefits of Ashwagandha phytochemicals, to determine the effect of withanolide A, using an in-vitro model of the effects of high serum 7KC on the BBB. The aim of the experiments is to elucidate the effects of withanolide A on 7KC-induced injury in hCMEC/D3 brain endothelial cells.

## 2. Materials and Methods

### 2.1. Cell Culture and Chemicals

The hCMEC/D3 human brain endothelial cell line was used in the present study. This cell line was derived from microvessels isolated from the human temporal lobe during excision of tissue for control of epilepsy. Isolates enriched in brain endothelial cells were immortalized by lentiviral vector transduction with the catalytic subunit of human telomerase (hTERT) and SV40 large T antigen. Cerebral endothelial cells were thereafter isolated by limited dilution cloning and extensively characterized for brain endothelial phenotype [24]. hCMEC/D3 cells were purchased from Merck Millipore, Burlington, MA, USA, and grown to confluence in T-75 Cell Culture Flasks (ThermoFisher, Waltham, MA, USA) coated with Rat Tail Collagen Type I (Sigma Aldrich, Burlington, MA, USA). The cells were cultured at 37 °C and 5% CO_2_, in media containing EBM 2 and EGM Plus Endothelial Bullet Kit (Lonza, Walkersville, MD, USA). All experiments were carried out on cell passages 5–30. A 400 μM stock solution of withanolide A was prepared by dissolving 1 mg of withanolide A (Cayman Chemical, Ann Arbor, MI, USA) in 200 μL of DMSO and diluted in 5.11 mL of milliQ water. The final concentration of DMSO when used in cell culture was <0.001%. A 1 mM stock solution of 7KC was prepared by dissolving 7KC (Cayman Chemical, Ann Arbor, MI, USA) in 5% ethanol (vehicle). A fixed concentration of 30 μM 7KC was used to induce cell death, based on previous dose-response experiments conducted in our laboratory [25].

### 2.2. MTS Cell Viability Assay

hCMEC/D3 cells were seeded in 96-well plates at a density of 1 × 10^4^ cells per well. CellTiter 96^®^ AQueous One Solution Cell Proliferation Assay (Promega, Madison, WI, USA) was used to determine the viability of these cells. After pretreatment with withanolide A for an hour, cells were treated with 7KC for 24 h to induce cell death and to test for protective effect of withanolide A [25]. Pilot experiments were carried out, where cells were pre-treated with 1 μM, 5 μM or 10 μM of withanolide A for one hour, followed by co-incubation with 30 μM of 7KC for 24 h. Based on preliminary results of cell-survival assays, 1 μM withanolide A was selected for subsequent experiments in this study, as it was the lowest concentration that showed a protective effect against 7KC cytotoxicity. Experiments were conducted with media only (untreated controls), 5% ethanol diluted in culture media (vehicle control), 1 μM withanolide A only, 30 μM 7KC only, and 30 μM 7KC added in combination to wells that have been pre-treated with 1 μM withanolide A. An additional condition of glucocorticoid receptor antagonist was added to some samples to test if the effect of withanolide A activity will be inhibited. These cells were incubated with 10 μM of the glucocorticoid receptor antagonist, mifepristone (RU486, Abcam, Cambridge, UK) in addition to withanolide A and 7KC. Each well was filled to a standard 100 μL with medium. Cells were left to grow in an incubator at 37 °C and 5% CO_2_ for 24 h. A mixture of medium and CellTiter 96^®^ AQueous One Solution Reagent was added to culture wells and incubated for 2 h before being analyzed using a 96-well plate reader at 490 nm. Colorimetric change of the solution occurs due to the reaction between reagent and cells, indicating cell viability/proliferation. Two wells were used for each treatment condition (technical replicates), and four separate experiments were carried out for the assay (biological replicates, *n* = 4).

### 2.3. Quantitative RT-PCR of Inflammatory Genes

Cells derived from T-75 cell culture flasks were seeded at 1 × 10^6^ per mL of medium in each well of 6-well plates and incubated at 37 °C in a humidified atmosphere containing 5% CO_2_ until they reached at least 70% confluence. Cells were then pre-treated with 1 µM withanolide A as there was significant rescue of cells from 7KC cytotoxicity at this concentration, followed by co-incubation with 30 μM 7KC for 24 h. Controls and vehicle controls were also included with similar conditions. RNA was extracted with the TRIzol (Thermo Fisher, Waltham, MA, USA) kit according to the manufacturer’s instructions. RNA concentration and purity was measured using NanoDrop 2000 (Thermo Fisher Scientific, Waltham, MA, USA). 1 μg of RNA was transcribed into cDNA using the High-Capacity cDNA Reverse Transcription Kit (Applied Biosystems, Waltham, MA, USA) at 25 °C for 10 min, 37 °C for 120 min, then 85 °C for 5 min, in the T-Personal Thermocycler (Biometra, Göttingen, Germany). The 7500 Real-time PCR System (Applied Biosystems, Waltham, MA, USA) was used to carry out real-time PCR amplification, using TaqMan Universal PCR Master Mix (Applied Biosystems, Waltham, MA, USA), with TaqMan Gene Expression Assay Probes for COX-2 (Hs00153133_m1), IL-1β (Hs01555410_m1), IL-6 (Hs00174131_m1), IL-8 (Hs00174103_m1), Nuclear Factor Kappa B (NF-κB) (Hs00765730_m1), TNF-α (Hs00174128_m1), and GAPDH (Hs02786624). The PCR reaction conditions were as follows: 95 °C for 10 min, 40 cycles of: 95 °C for 15 s and 60 °C for 1 min. Two wells were used for each treatment condition, and four separate experiments were carried out for the assay.

### 2.4. COX-2 Enzyme Activity Assay

The enzyme COX-2 is a key enzyme in the inflammatory pathway, as it metabolises arachidonic acid to prostaglandins and other proinflammatory mediators. The expression of COX-2 is induced by cytokines [26], including IL-1β [27] and TNFα [28]. Cells were cultured in 6-well plates as described above. They were pre-treated with 1 μM withanolide A before treatment with 30 μM 7KC along with untreated- and vehicle controls. An additional condition of glucocorticoid receptor antagonist was added to some samples to test if the effect of withanolide A activity will be inhibited. These cells were incubated with 10 μM of the glucocorticoid receptor antagonist, mifepristone (Abcam, Cambridge, UK) in addition to withanolide and 7KC. Cyclooxygenase (COX) Activity Assay Kit (Fluorometric) (ab204699, Abcam, Cambridge, UK) was used to detect the peroxidase activity of COX, with COX-1 and COX-2 specific inhibitors to differentiate the activity of COX-1 and COX-2 as well as other peroxidases which may be present in the sample, to accurately determine if COX-2 activity in cells is increased. The assays were carried out according to the manufacturer’s instructions. Detection of COX activity was done using a microplate reader. Two wells were used for each treatment condition, and four separate experiments were carried out for the assay.

### 2.5. ROS Assay

The level of ROS in cells was quantified using DCFDA (Invitrogen, Waltham, MA, USA), a dye that fluoresces green when bound to ROS. Cells were cultured in 6-well plates, detached, and cell pellets were washed twice with 1 X PBS. They were incubated for 30 min with H2DCFDA in the absence of light, before conducting flow cytometry analysis. The latter was performed using a Cytoflex LX flow cytometer (Beckman Coulter Life Sciences, Chaska, MN, USA), with 1 × 10^6^ cells per sample for analyses. A total of 10,000 cells per sample was recorded. The FL1 channel was used to quantify ROS (DCFDA, Ex/Em = 495/527 nm).

### 2.6. Quantitative PCR of Clotting Genes

Procedures were identical to those described for inflammatory genes, except those genes tested were FII/ Thrombin (Hs01011988_m1), von Willebrand factor/VWA (Hs01651043_m1), Thromboxane A Synthase/TBXAS1 (Hs01022705_m1), Factor 8/FVIII (Hs00252034_m1), inducible nitric oxide synthase (iNOS)/NOS2 (Hs01075529_m1), and endothelial nitric oxide synthase (eNOS)/NOS (Hs01574665_m1). GAPDH (Hs02786624) was used as the housekeeping gene. The PCR reaction conditions were as follows: 95 °C for 10 min, 40 cycles of: 95 °C for 15 s and 60 °C for 1 min. Two wells were used for each treatment condition, and four separate experiments were carried out for the assay.

### 2.7. Thrombin Enzyme Activity Assay

Cells were cultured in 12-well plates at a density of 1 × 10^5^ cells per well. They were pre-treated with 1 μM withanolide A before treatment with 30 μM 7KC, along with vehicle controls. An additional condition of glucocorticoid receptor antagonist was added to some samples to test if the effect of withanolide A activity will be inhibited. These cells were incubated with 10 μM of mifepristone in addition to withanolide A and 7KC. Thrombin Activity Assay kit (ab234621, Abcam, Cambridge, UK) was used according to the protocol provided, to determine the level of enzyme activity of thrombin, and confirm whether increased gene expression of thrombin is translated to increased enzymatic activity. Two wells were used for each treatment condition, and four separate experiments were carried out for the assay.

### 2.8. Statistical Analysis

All data was first entered into Microsoft Excel 365. GraphPad Prism 6 was used for significance testing using one-way ANOVA, followed by Bonferroni’s multiple comparison post-hoc test. Results were considered statistically significant at *p* < 0.05.

## 3. Results

### 3.1. MTS Cell Viability Assay

**Figure 1:**
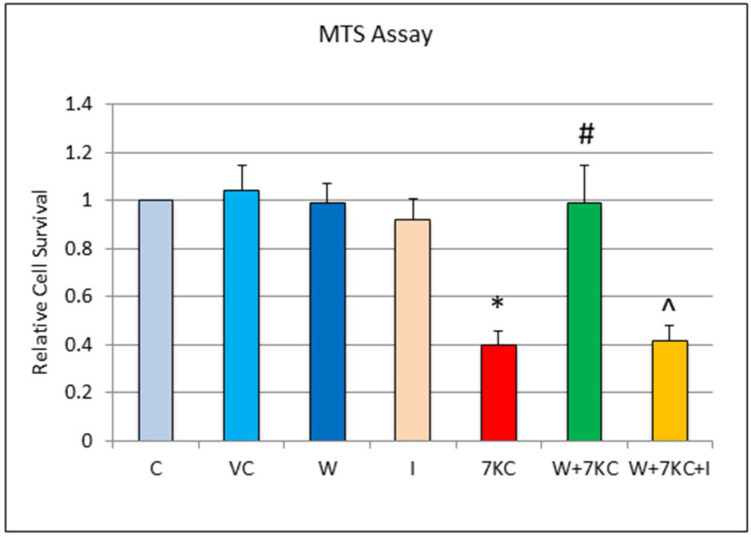
Effect of withanolide A on 7KC-induced cytotoxicity, determined by MTS assay. hCMEC/D3 cells were treated with vehicle only, 7KC only, withanolide A only, pre-treated with 1 μM withanolide A for 1 h, followed by co-incubation with 30 μM 7KC for 24 h or pre-treated with 1 μM withanolide A plus 10 μM mifepristone for 1 h, followed by co-incubation with 30 μM 7KC for 24 h. Co-treatment of cells with withanolide A results in reduction in 7KC-induced cytotoxicity. The protective effect of withanolide A was abolished when cells were co-treated with withanolide A and mifepristone plus 7KC. C: Control, VC: Vehicle control, W: 1 μM withanolide A, I: 10 μM mifepristone, 7KC: 30 μM 7KC. W+7KC indicates co-treatment of withanolide A plus 7KC. W+7KC+I indicates co-treatment of withanolide A plus 7KC plus mifepristone. Data are represented as mean ± SD (*n* = 4). * Significant difference compared to controls. # Significant difference compared to 7KC ^ Significant difference compared to withanolide A + 7KC (all *p* < 0.05).

A significant reduction in the cell viability of hCMEC/D3 endothelial cells was found by MTS assay, 24 h after the treatment with 7KC. The loss of cell viability was significantly prevented by treatment of cells with withanolide A. This protection was abolished when cells were treated with withanolide A and 10 μM of the glucocorticoid receptor antagonist, mifepristone, together with 7KC (Figure 1).

### 3.2. Quantitative RT-PCR of Inflammatory Genes

**Figure 2:**
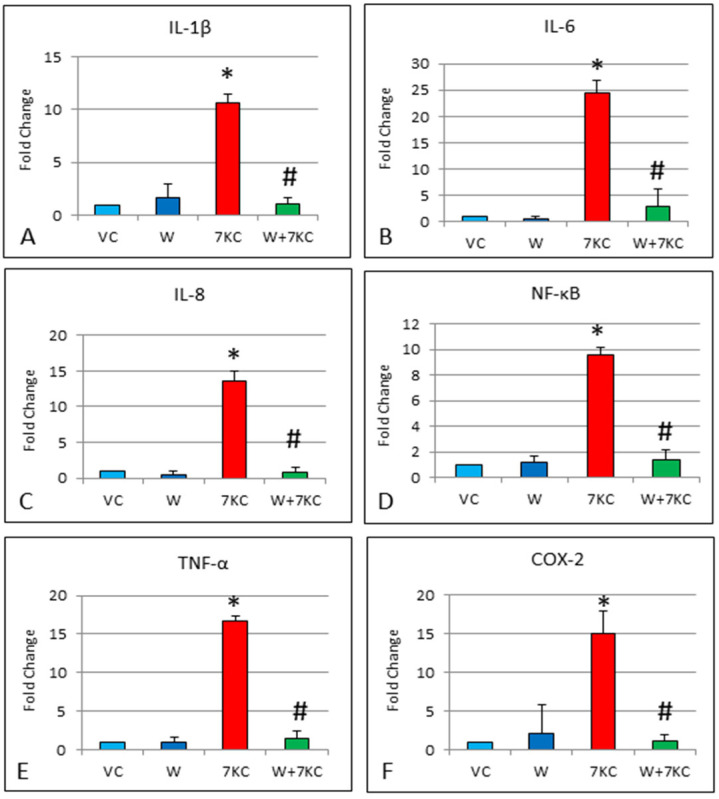
Effect of withanolide A on 7KC-induced increase in mRNA expression of pro-inflammatory genes, determined by real time RT-PCR. hCMEC/D3 cells were treated with vehicle only, 7KC only, withanolide A only, or pre-treated with 1 μM withanolide A for 1 h, followed by co-incubation with 30 μM 7KC for 24 h (**A**–**F**). Treatment of cells with 7KC resulted in significant induction of (**A**–**F**) IL-1β, IL-6, IL-8, NF-κB, TNF-α and COX-2 mRNA expression. These increases were significantly reduced by co-treatment with withanolide A. VC: Vehicle, W: 1 μM withanolide A, 7KC: 30 μM 7KC. Data are represented as mean ± SD (*n* = 4). * Significant difference compared to controls. # Significant difference compared to 7KC (all *p* < 0.01).

Endothelial cells that have been treated with 7KC showed significant increase in mRNA expression of IL-1β, IL-6, IL-8, NF-κB, TNF-α, and COX-2, 24 h after treatment with 7KC. IL-6 showed the greatest increase at 24-fold change, followed by TNF-α at 17 fold, and COX-2 at 15 fold change. The increases in inflammatory gene expression were significantly reduced by treatment of cells with withanolide A (Figure 2).

### 3.3. COX-2 Assay

**Figure 3:**
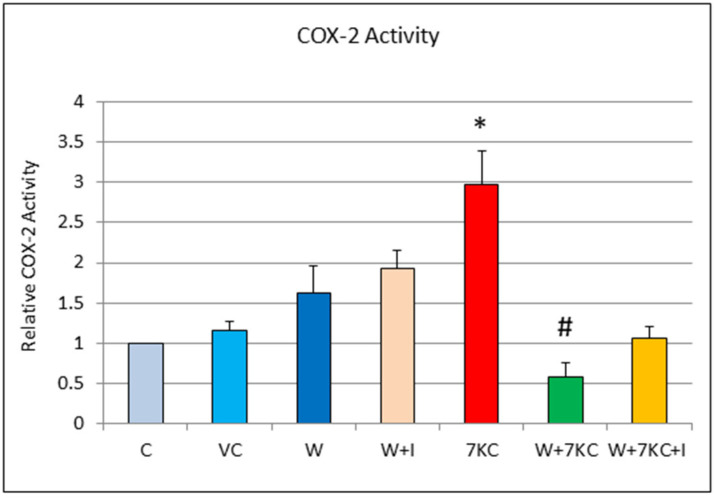
Effect of withanolide A and 7KC on COX-2 enzymatic activity. Significant increase in COX-2 activity was observed after treatment of cells with 7KC. This increase was significantly reduced by withanolide A. The modulatory effect of withanolide against increase in COX-2 activity was partially reduced (non-significant) when the cells were co-treated with withanolide A and mifepristone plus 7KC. C: Control, VC: Vehicle control, 7KC: 30 μM 7KC, W: 1 μM withanolide I: 10 μM mifepristone. Data are represented as mean ± SD (*n* = 4). * Significant difference compared to controls. # Significant difference compared to 7KC (both *p* < 0.01).

A significant increase in COX-2 activity was found in homogenates of hCMEC/D3 endothelial cells, 24 h after treatment with 7KC. The increase in COX-2 activity was significantly reduced by co-incubation of cells with withanolide A. This effect was partially reduced (though non-significant), when cells were treated with withanolide A plus 10 μM mifepristone, together with 7KC (Figure 3).

### 3.4. ROS Assay

**Figure 4:**
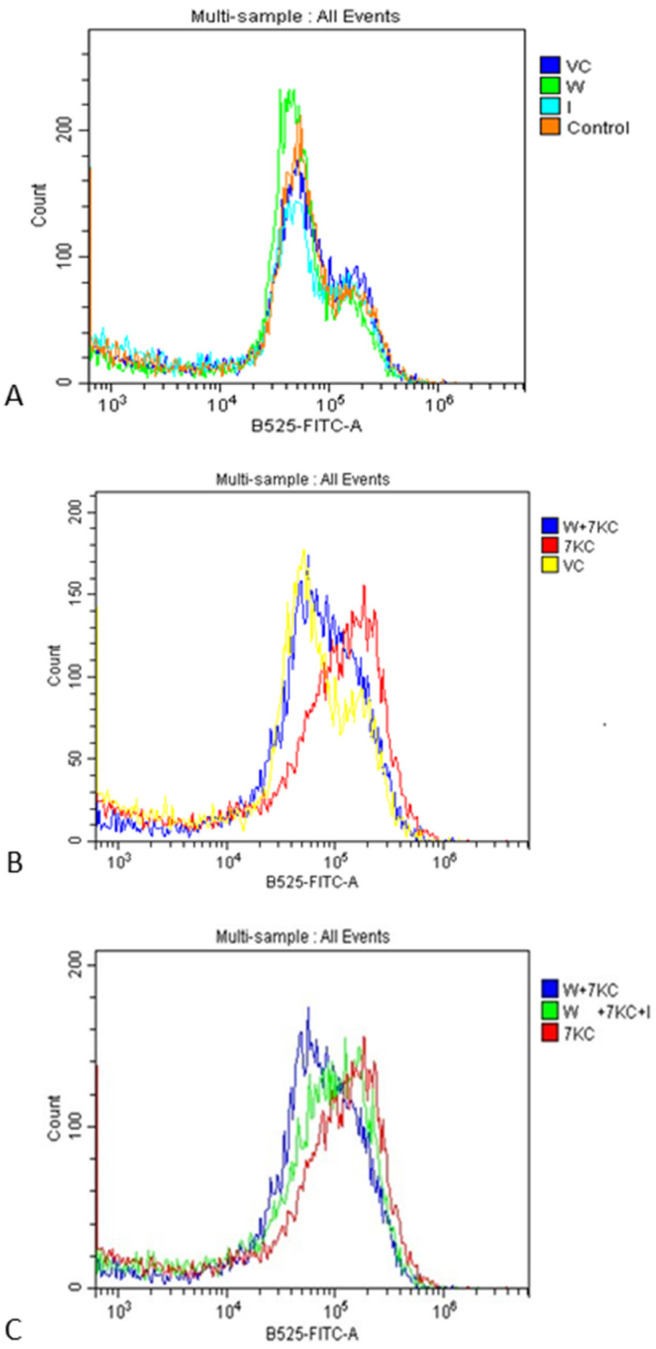
(**A**–**C**) Single parameter histograms showing comparison between various treatment groups in terms of fluorescence intensity for DCFDA. *X* axis indicates fluorescence intensity, detected at 525 nm. *Y* axis indicates the number of cells. A rightward shift of the curve indicates a greater number of cells with higher intensity, i.e., greater levels of ROS. A: blue line: Vehicle control (VC). Green line: withanolide A only (W). Turquoise line: mifepristone only (I). Red line: untreated control. No difference from untreated control or vehicle control is seen with withanolide A treatment, or mifepristone treatment only. (**B**,**C**): Yellow line: Vehicle control; Red line: 7KC only; Blue line: withanolide A with 7KC co-incubation; Green line: withanolide A with mifepristone and 7KC co-incubation. Withanolide A has an effect in reducing ROS formation by 7KC, and this effect was abrogated by mifepristone.

No change in fluorescence intensity was seen after addition of withanolide A or mifepristone alone, compared to untreated controls or vehicle controls (overlapping plots in Figure 4A). 7KC-only treatment induced an increase in DCFDA fluorescence intensity (right shift in fluorescence intensity in red plot, in Figure 4B) as compared to vehicle control, indicating increased ROS levels in cells. Withanolide A attenuated the increase in ROS production when co-incubated with 7KC (Figure 4B). The addition of mifepristone abolished the protective effect of withanolide A against 7KC-induced ROS production (Figure 4C).

### 3.5. Quantitative RT-PCR of eNOS and iNOS, and Clotting Genes

**Figure 5:**
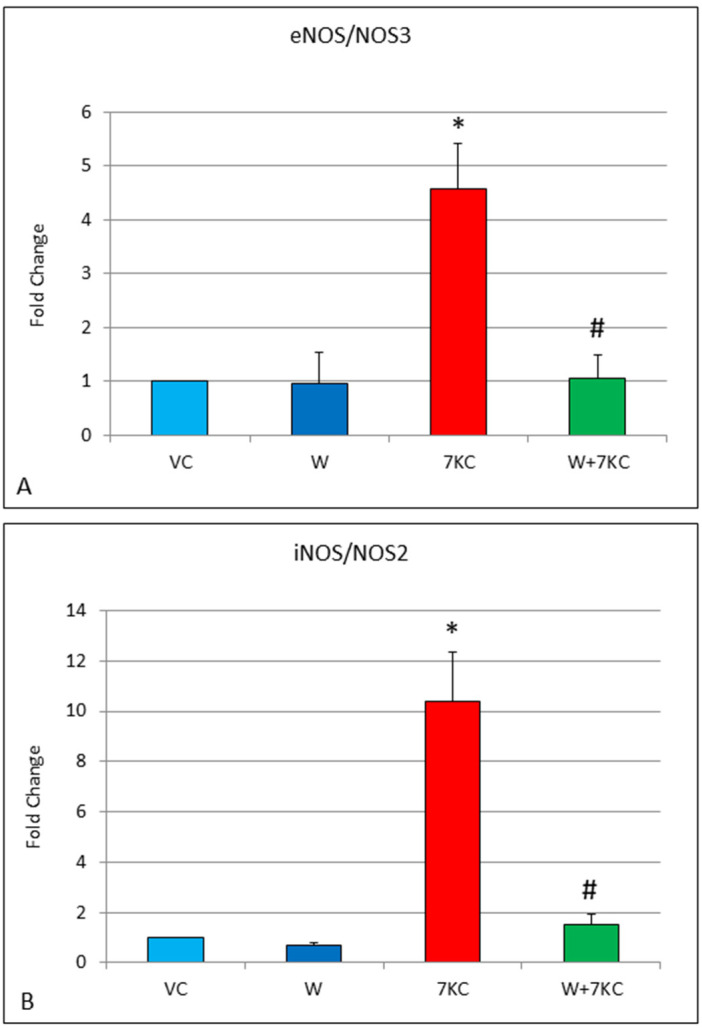
(**A**,**B**): Effect of withanolide A and 7KC on eNOS and iNOS mRNA expression, determined by real time RT-PCR. A 4.6-fold increase in eNOS, and a 10.4-fold increase in iNOS was found after treatment of endothelial cells with 7KC. The increases were significantly reduced by withanolide A. Data are represented as mean ± SD (*n* = 4). * Significant difference compared to controls. # Significant difference compared to 7KC (all *p* < 0.01).

**Figure 6:**
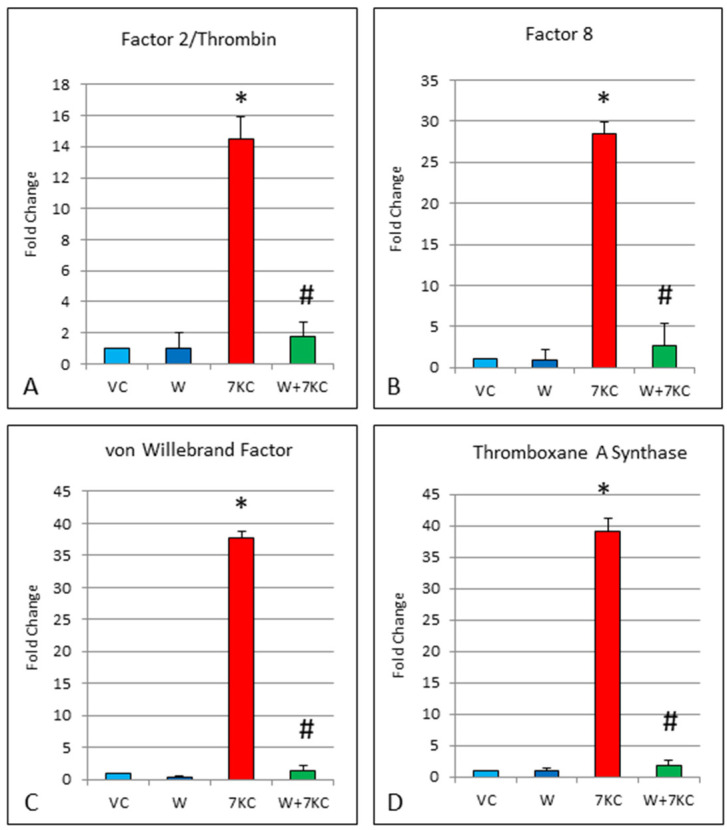
(**A**–**D**) Effect of withanolide A on 7KC-induced increase in mRNA expression of clotting genes, determined by real time RT-PCR. hCMEC/D3 cells were pre-treated with 1 μM withanolide A for 1 h, followed by co-incubation with 30 μM 7KC for 24 h. Treatment of cells with 7KC resulted in significant induction of Factor 2/thrombin, Factor 8, von Willebrand factor and thromboxane A synthase mRNA expression. These increases were significantly reduced by withanolide A. Data are represented as mean ± SD (*n* = 4). * Significant difference compared to controls. # Significant difference compared to 7KC (all *p* < 0.01).

Endothelial cells that had been treated with 7KC showed a 4.6-fold increase in eNOS mRNA expression, and a larger 10.4-fold increase in iNOS mRNA expression, 24 h after treatment with 7KC. The increases in eNOS and iNOS were significantly reduced by co-incubation of cells with withanolide A (Figure 5). Likewise, treatment of endothelial cells with 7KC resulted in significant induction of Factor 2/thrombin, Factor 8, von Willebrand factor and thromboxane A synthase mRNA expression, and these increases were significantly attenuated by co-incubation with withanolide A (Figure 6).

### 3.6. Thrombin Activity Assay

**Figure 7:**
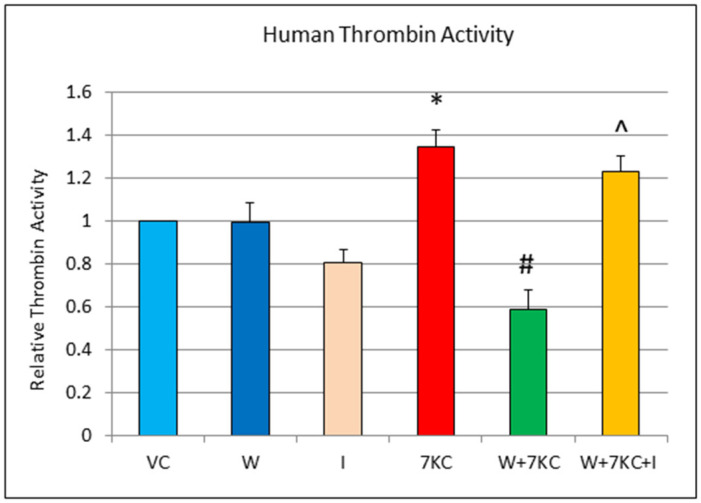
Effect of withanolide A and 7KC on human thrombin activity. Significant increase in thrombin activity was observed after treatment of cells with 7KC. This increase was significantly reduced by withanolide A. The modulatory effect of withanolide A against increase in 7KC induced thrombin activity was abolished when cells were co-treated with withanolide A and mifepristone together with 7KC. Data are represented as mean ± SD (*n* = 4). * Significant difference compared to controls. # Significant difference compared to 7KC ^ Significant difference compared to withanolide A + 7KC (all *p* < 0.01).

A significant increase in thrombin activity was found in homogenates of hCMEC/D3 endothelial cells using the assay kit, 24 h after treatment with 7KC. This increase in activity was significantly reduced by the co-incubation of cells with withanolide A. The modulatory effect was abolished when cells were treated with withanolide A and 10 μM mifepristone, together with 7KC (Figure 7).

## 4. Discussion

This study was carried out to determine the effect of a well-established component of Ashwagandha (*Withania somnifera*; Solanaceae), withanolide A on 7KC induced endothelial injury. 7KC and 7β-hydroxycholesterol cause a specific form of cytotoxic activity known as oxiapoptophagy [6] and show potent cytotoxic effects on bovine aortic endothelial cells [7]. Moreover, 7KC induces downregulation of the adherens junction protein VE-cadherin, but upregulation of α-smooth muscle actin, fibroblast activation protein and transforming growth factor beta, and produces an increase in cell migration in choroidal endothelial cells [29]. A high-fat diet has been found to disrupt endothelial reendothelialization after vascular injury in mice; and this is likely related to 7KC- induced activation of the notch1 signaling pathway and endothelial cell senescence [30]. Cholesterol derivatives in oxidized low-density lipoprotein such as 7KC, 7α-hydroxycholesterol, and 7β-hydroxycholesterol have an important effect in reducing endothelium-dependent arterial relaxation of rabbit aortic rings. This is not the result of oxidation of nitric oxide (NO) by the superoxide radical, but rather, the effect of protein kinase C signaling in endothelial cells [31]. Withanolide A has previously been found to protect against oxidative and DNA damage-stress caused by hydrogen peroxide in neuroblastoma and glioblastoma cells [32]. In this study, withanolide A was also demonstrated to have a protective effect against cell death and inflammation induced by 7KC in brain endothelial cells. We confirmed the results of previous studies which have found increases in pro-inflammatory cytokines IL-1β, IL-6, IL-8 and TNFα after exposure to 7KC [33,34,35]; and showed that these increases were significantly reduced by withanolide A. These findings indicate a significant effect of withanolide A against 7KC induced cytotoxicity and inflammatory gene induction in brain endothelial cells.

A significant [26,27,28] increase in COX-2 mRNA expression and enzymatic activity was detected in hCMEC/D3 brain endothelial cells after treatment with 7KC, and this increase was reduced by withanolide A. COX-2 converts arachidonic acid that is released by cytosolic phospholipase A_2_ (cPLA_2_) to prostaglandins. Further metabolism of prostaglandins produces free radicals that could contribute to oxidative stress [36]. Flow cytometry analysis using the ROS indicator, DCFDA, confirms that 7KC increases ROS production, and this increase is reduced by withanolide A. Moreover, nitrosative stress could occur via an increase in NO production [36], and 7KC was found to significantly increase the expression of iNOS, and to a lesser extent, eNOS. These increases were attenuated by withanolide A. Collectively, the above results indicate cytoprotective, anti-inflammatory, and anti-oxidative effects of withanolide A against 7KC injury in brain endothelial cells.

Previous studies have shown that withanolide A treatment exerts a neuroprotective- and anti-neuroinflammatory effect in the hippocampus after pilocarpine-induced status epilepticus [37]. Withanolide A was found to suppress lipopolysaccharide-induced increase in inflammatory responses, NO production and ROS formation in BV-2 microglial cells through induction of the Nrf2-heme oxygenase-1 pathway, which leads to the transcription of antioxidant genes [17]. Withanolide A also reduced lipopolysaccharide-induced expression of inflammatory cytokines in bone marrow derived macrophages via inhibition of the MAPK and NF-kB pathways [38]. Furthermore, withanolide A was found to protect against glutamate-mediated excitotoxicity via the PI3K/Ak/MAPK signalling pathway [39].

Besides inflammatory genes, RT-PCR of blood clotting genes was carried out, as there is a proposed link between inflammation and the clotting cascade [40]. Factor 2/thrombin, Factor 8, von Willebrand factor, and thromboxane A synthase showed significant increase in gene expression after 7KC treatment, but these increases were significantly reduced by withanolide A. Confirmation of the gene expression results was done using the thrombin activity assay. Results showed that 7KC induced increased thrombin enzymatic activity, and this increase was reduced by withanolide A.

Relatively little is known about the mechanisms behind the anti-inflammatory and anti-clotting effects of withanolide A, although it has been reported that a structurally related compound, ginsenoside, exerts its effect partly through binding to the glucocorticoid receptor [41,42]. Ginsenosides are functional ligands of glucocorticoid receptors and appear to inhibit kinase phosphorylation including MAPK and ERK1/2, NF-κB transcription factor induction/translocation, and DNA binding [43,44]. Since ginsenoides (from Korean ginseng) and withanolides (from Indian ginseng) have similar chemical structures, we sought to determine whether the glucocorticoid receptor could be important for the cellular effects of withanolide A. Interestingly, the protective effects of withanolide A against cytotoxicity, ROS production and increased thrombin activity produced by 7KC were abrogated, when the glucocorticoid receptor antagonist, mifepristone was added together with withanolide A. These results suggest that the glucocorticoid receptor could play a role in the cytoprotective, anti-oxidative and anti-clotting effects of withanolide A.

Withanolide A increases glutathione biosynthesis in neuronal cells in a corticosterone-dependent manner [23]. Profiling of withanolide A for therapeutic targets in neurodegenerative diseases has shown that withanolide A forms several contacts with residues in the glucocorticoid binding pocket but lacks key stabilizing interactions as observed for dexamethasone [45]. More studies are therefore needed to elucidate the possible mechanisms of action of withanolide A, on the glucocorticoid receptor.

Variants in the cholesterol-binding protein, apoE have been suggested to increase the risk of cardiovascular disease and Alzheimer’s disease [46,47]. There is also evidence that apoE mutations may affect BBB function [48]. It is possible that strategies to reduce cholesterol oxidation or decrease the cytotoxic effect of cholesterol oxidation products could help prevent/reduce damage to brain endothelial cells, thromboembolic events such as ischemic stroke, and damage to the BBB.

Together, the above results indicate that withanolide A increases cell survival, reduces inflammatory gene expression and COX-2 activity and reduces ROS in brain endothelial cells that have been treated with 7KC. Moreover, withanolide A reduces the 7KC-induced increases in clotting factors expression and thrombin activity, which is one of the last steps in the clotting cascade. Further studies are necessary to elucidate the detailed mechanisms of action of withanolide A against oxysterol-induced injury.

## Data Availability

Not applicable.
